# The diversity of animals identified as keystone species

**DOI:** 10.1002/ece3.10561

**Published:** 2023-10-09

**Authors:** Ishana Shukla, Kaitlyn M. Gaynor, Boris Worm, Chris T. Darimont

**Affiliations:** ^1^ Department of Geography University of Victoria Victoria British Columbia Canada; ^2^ Departments of Botany and Zoology University of British Columbia Vancouver British Columbia Canada; ^3^ Department of Biology Dalhousie University Halifax Nova Scotia Canada; ^4^ Raincoast Conservation Foundation Denny Island British Columbia Canada

**Keywords:** community ecology, ecosystem engineer, indirect effects, interaction strength, keystone species

## Abstract

Although the keystone species concept was conceived of over 50 years ago, contemporary efforts to synthesize related literature have been limited. Our objective was to create a list of keystone animal species identified in the literature and to examine the variation in the traits of species and the ecosystem influences they elicit. We documented 230 species considered keystones. A clustering analysis classified them into five archetypes based on combinations of their taxonomic class, body size, trophic level, and role (consumers, modifiers, or prey). Although conservation and public perception of keystones primarily focuses on large vertebrate consumers, our analysis reveals that researchers have defined a wide diversity of keystone species, with large variation in associated ecosystem processes. Future research may confront ambiguity in the definition of keystone status, as well as clarify the type, abundance, and quality of data required to assign the term. Identifying keystones with increased rigor would not only enrich the literature but also inform intervention to safeguard threatened keystones and their associated influences on ecosystems.

## Graphical abstract

Although the keystone species concept was conceived of over 50 years ago, contemporary efforts to synthesize related literature have been limited. We compiled a database of 230 keystone vertebrates and invertebrates, and through a clustering analysis, we revealed five primary keystone ‘archetypes’. Although conservation and public perception of keystones primarily focuses on large vertebrate consumers, our analysis reveals a wide diversity of keystone species, with large variation in associated ecosystem processes.

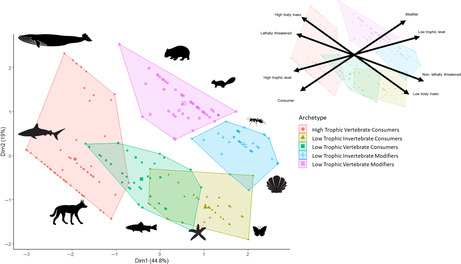

## 1 INTRODUCTION

The ‘keystone species’ concept, coined by Paine (1969), originally referred to a single species that made ‘great modification’ to the species composition or appearance of an ecosystem. The concept has remained prominent in ecological research, education, and conservation for decades. Contemporary efforts to synthesize knowledge, however, have been limited. Multiple definitions and functions of keystone species exist, with each definition expanding the keystone species concept from predator, to include prey, ecosystem modifiers, and beyond (e.g., Cantor & Whitham, 1989; Mills et al., 1993; Paine, 1995; Power et al., 1996). However, these definitions have yet to be considered collectively. Moreover, the taxa associated with evidence for keystoneness has likewise not been summarized. Meanwhile, cultural and conservation perception often focuses on a narrow view of keystones—primarily oriented toward large terrestrial carnivores. Wolves (*Canis lupus*) and other large‐bodied terrestrial top carnivores are often credited with sweeping community and landscape effects via their roles as predators and imposing behavioral changes in the community (Ripple et al., 2014, *but see* Gable et al., 2020).

Against this background, we present here a comprehensive summary and descriptive analysis of keystones species, as identified by others in the relevant literature. Our objective was to synthesize the related body of work among animal taxa, focusing on documented assertions of keystone species, the traits they possess, and the responses they invoked in ecological communities. Notably, although we clearly define the criteria we use to detect relevant literature on keystones, we relied on the authors of original studies to identify species as keystones. Specifically, we accepted the assertions the authors provided for keystone designation. We comment generally, however, on the criteria and associated evidence these authors brought to bear in such designations. We conclude by identifying considerable variation among identified keystones, with overarching patterns that support the designation of five keystone ‘archetypes’.

## 2 METHODS

### 2.1 Documenting keystone species identified in the literature

**FIGURE 1:**
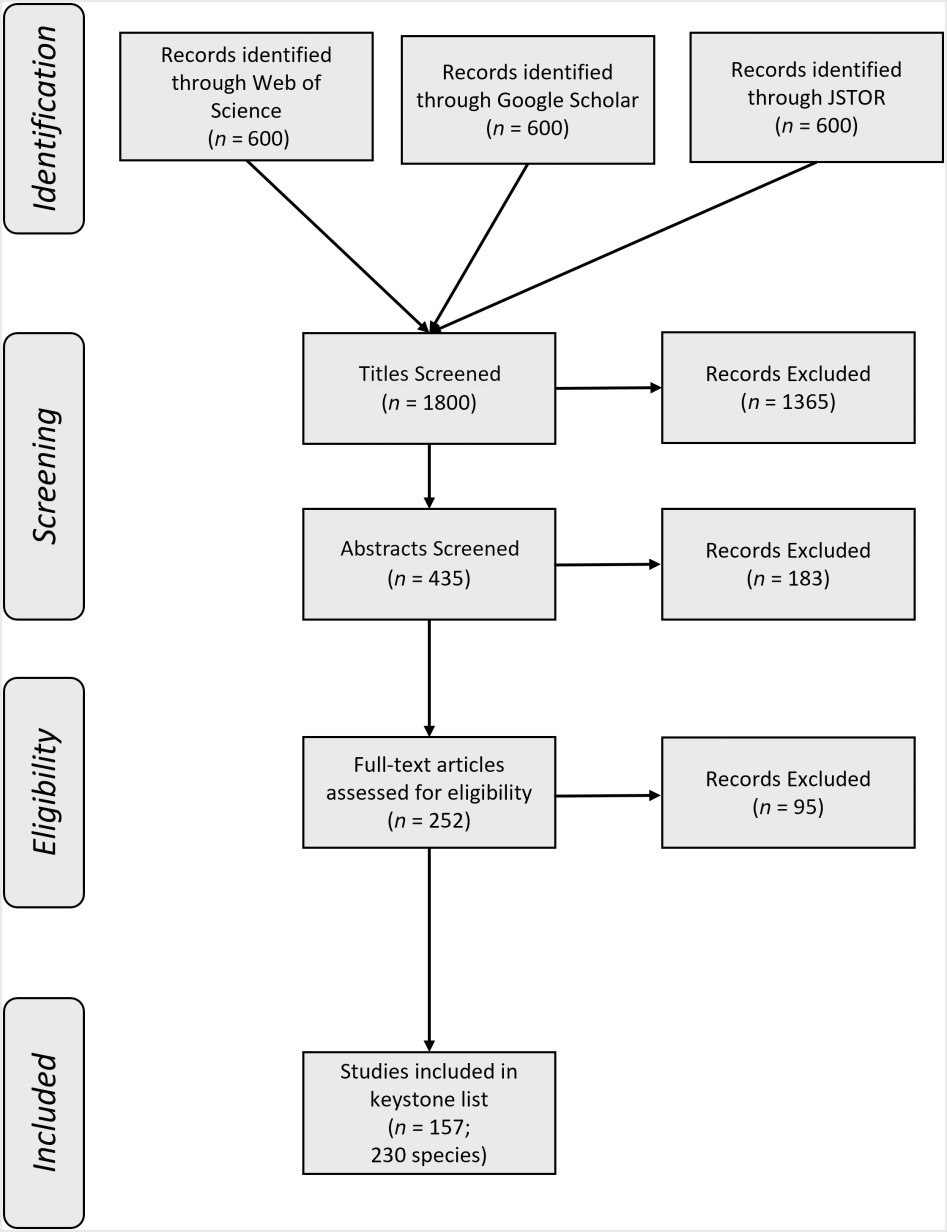
PRISMA (Preferred Reporting Items for Systematic Reviews and Meta‐Analyses) diagram depicting the selection criteria process for documenting animal species identified as keystones in the literature.

We conducted a systematic literature search to encompass and extract data on keystone species identified by other scientists (Moher et al., 2015). Here, our objective was to capture an existing landscape of keystone literature. We searched Google Scholar, Web of Science, and JSTOR with the search terms: keystone species OR keystone effect* OR keystone in the title or abstract. We read the first 600 titles from each database (*n* = 1800), and if the title was relevant, we read the abstract. If the title or abstract included one or more of our search terms, we scanned the full publication to determine whether it met our eligibility criteria: it had to (i) clearly refer to the species as a keystone or key species in the community and (ii) provide primary or secondary experimental or observational evidence for this assertion. We included the phrase ‘key species’ in our search during this preliminary scan because some publications that the original search yielded (*n* = 14) only provided another study for reference; in such cases, we defaulted to the cited study (that used the term ‘key species’) as a source. These originally cited studies rarely labeled the species as a keystone and instead only stated that they were low in abundance relative to the disproportionately large influence it exerted in the ecosystem. If a species was represented more than once, we included the study that provided empirical evidence and that was the most recent. If the paper did not meet one or more of the criteria but still referred to the species as a keystone, we performed a backward search to attempt to identify the source paper(s) it cited in the context of referring to their role as keystone species (or ‘keystoneness’; Hurlbert, 1997). Finally, we located additional publications not found by our search (*n* = 14) by inspecting titles of references within included papers (Figure 1). Identified keystones originated from 94 peer‐reviewed journals, with the most represented journals (36%) being Ecology (*n* = 13), Science (*n* = 11), and Frontiers in Ecology and the Environment (*n* = 10). Journal Impact Factors (2023) ranged from 0.36 to 47.7 and had a mean of 7.7 (Garfield, 1999).

**FIGURE 2:**
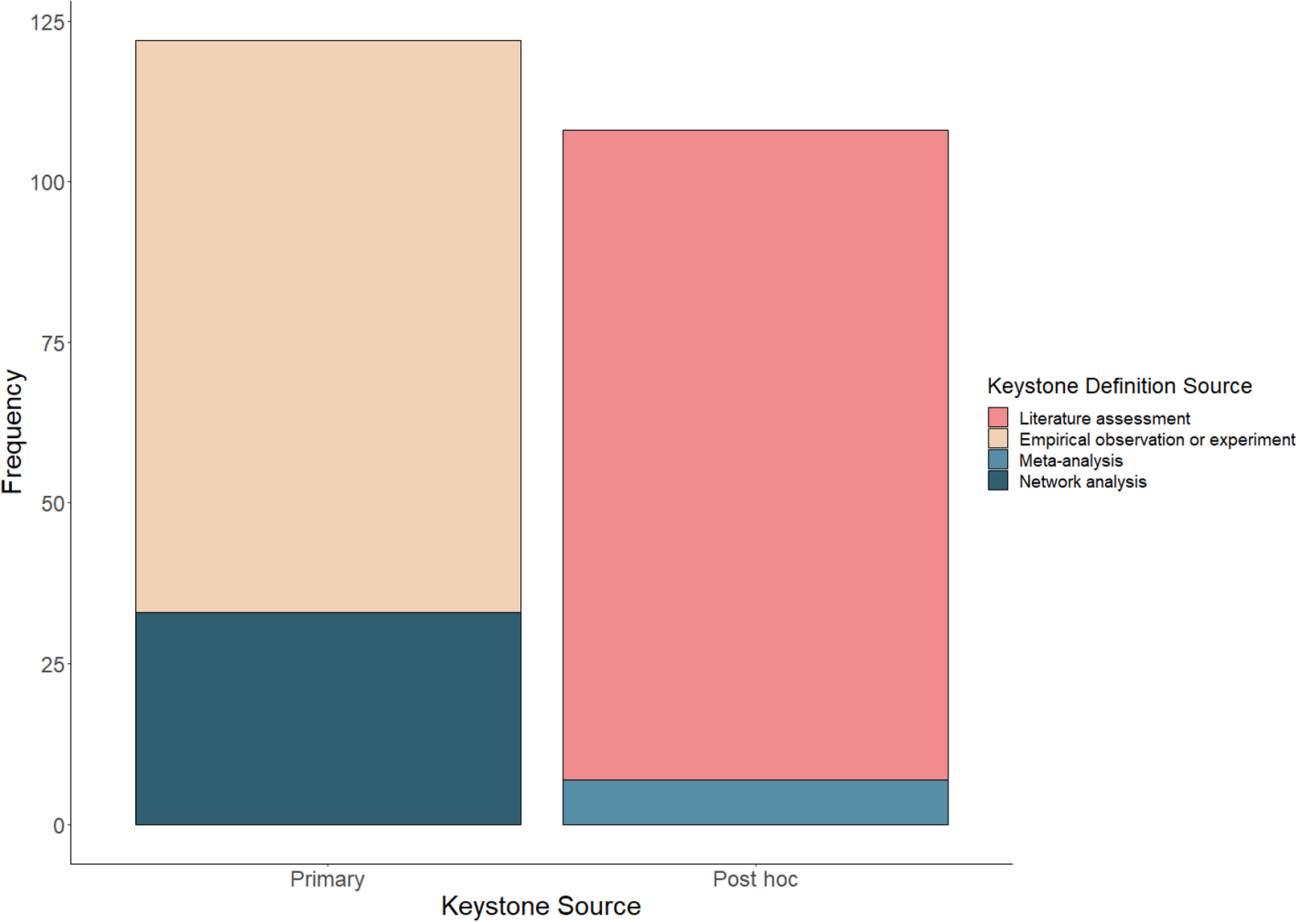
Justifications for keystoneness as provided by authors of original studies (*n* = 230 species).

We also categorized the rationale for keystone status provided by the authors (Figure 2). ‘Primary’ referred to any keystone identified by a study (experimental or observational) that included primary evidence of a change in community response (i.e., a significant change in abiotic or biotic factors in response to a gradient of keystone density, or presence versus absence; *n* = 123). ‘Primary’ also referred to a species sourced from a network analysis that found evidence of keystoneness (sensu Libralato et al., 2006) in which the keystoneness of a species is measured as a function of its modeled ecosystem impact relative to its biomass. A classification as ‘Post hoc’ referred to any species for which keystone status was assigned by authors after they assessed evidence from two or more empirical studies (*n* = 107 species). Post hoc also included meta‐analyses, studies that assign keystone status to multiple species based on multiple quantitative sources (*n* = 5 species; Myers et al., 2007).

### 2.2 Data extraction

We extracted the following data from identified studies to create a comprehensive list that we subjected to a clustering analysis informed by species' traits and ecosystem effects. We recorded the keystone species' role in the ecosystem (i.e., consumer, prey, or modifier), body mass, and trophic level, as well as the community response to the keystone's absence. We also classified their habitat (aquatic or terrestrial) and taxonomic class. For simplicity, we categorized taxonomic classes *Chondrichthyes* and *Actinopterygii* as ‘fish’, *Branchiopoda*, *Insecta*, and *Malacostraca* as ‘arthropods’, *Echinoidea*, *Asteroidea*, and *Ophiuroidea* as ‘echinoderms’, *Reptilia* and *Amphibia* as ‘herps’, and *Cephalopoda*, *Gastropoda*, *Bivalvia*, and *Polychaeta* as ‘molluss’. We classified roles as consumer (impacts the system through consumption), prey (directly sustains one or more predator populations), or modifier (significantly alters the landscape or habitat; Jones et al., 1994; Mills et al., 1993). We extracted average adult body mass from Fishbase, AmphiBIO, and AVONET (Froese & Pauly, 2010; Oliveira et al., 2017; Tobias et al., 2022).

We assigned a concise description for each type of community response, noting if multiple responses were reported. ‘Abundance’ referred to increases or decreases in the biomass or number of other species in the community (e.g., total biomass of a prey species). ‘Behavioral’ referred to changes to behavior that alter the spatiotemporal distribution of a species (e.g., significant changes in habitat use). ‘Biodiversity’ accounted for changes to richness, diversity, or community composition (e.g., total number of species). ‘Chemical and Energy Cycling’ referred to changes to nutrient, biogeochemical, or energy cycling in the affected ecosystem (e.g., percentage of labile carbon in soil). ‘Life history’ referred to a response in growth or reproduction among other species in the community (e.g., ratio of breeding pairs, changes in body size, breeding age/rate, etc.). Finally, ‘Physical effects’ included significant physical changes to the keystone's environment (e.g., dammed rivers, changes in sediment loads, habitat creation, etc.).

### 2.3 Clustering analysis

We described patterns among keystones based on their characteristics and influence in their environments, organizing them into ‘archetypes’. Using a *k*‐means clustering analysis, we dummy‐coded species characteristics, including taxonomic class, keystone role, trophic level, and also recorded (log standardized) body mass. For clustering analyses, we grouped herbivores, omnivores, and planktivores into a ‘low’ trophic level. Omnivores were classified as low trophic level because most were small‐bodied insectivores. Mesopredators were labeled as ‘mid’ trophic level, and secondary, tertiary, and apex predators were grouped in a ‘high’ trophic level. Visual inspection using the elbow method (Kumar et al., 2022) revealed the most prominent bend at *k* = 5, identifying the optimal number of clusters. This method aims to identify the number of clusters (*k*) with the smallest sum of square distances. The smallest value (or ‘bend’ in the graph) indicates the lowest sum of square distances. Given that this bend was not distinct, however, we also conducted multiple silhouette analyses (Kodinariya & Makwana, 2013) and selected the *k* with the largest width and the fewest negative values. Silhouette evaluation calculates the similarity of an individual data point to its assigned cluster, as compared to the distance to the other clusters. Generally, a higher average silhouette score indicates a better clustering, with 1 indicating a perfect match, and −1 indicating a perfect mismatch. Here, an average silhouette width of 0.31 (and only one negative width) indicated the five groups provided appropriate clustering. Once clusters were established, we drew on the geometry of the five vectors to qualitatively describe and label each group, or ‘archetype’.

## 3 RESULTS

**FIGURE 3:**
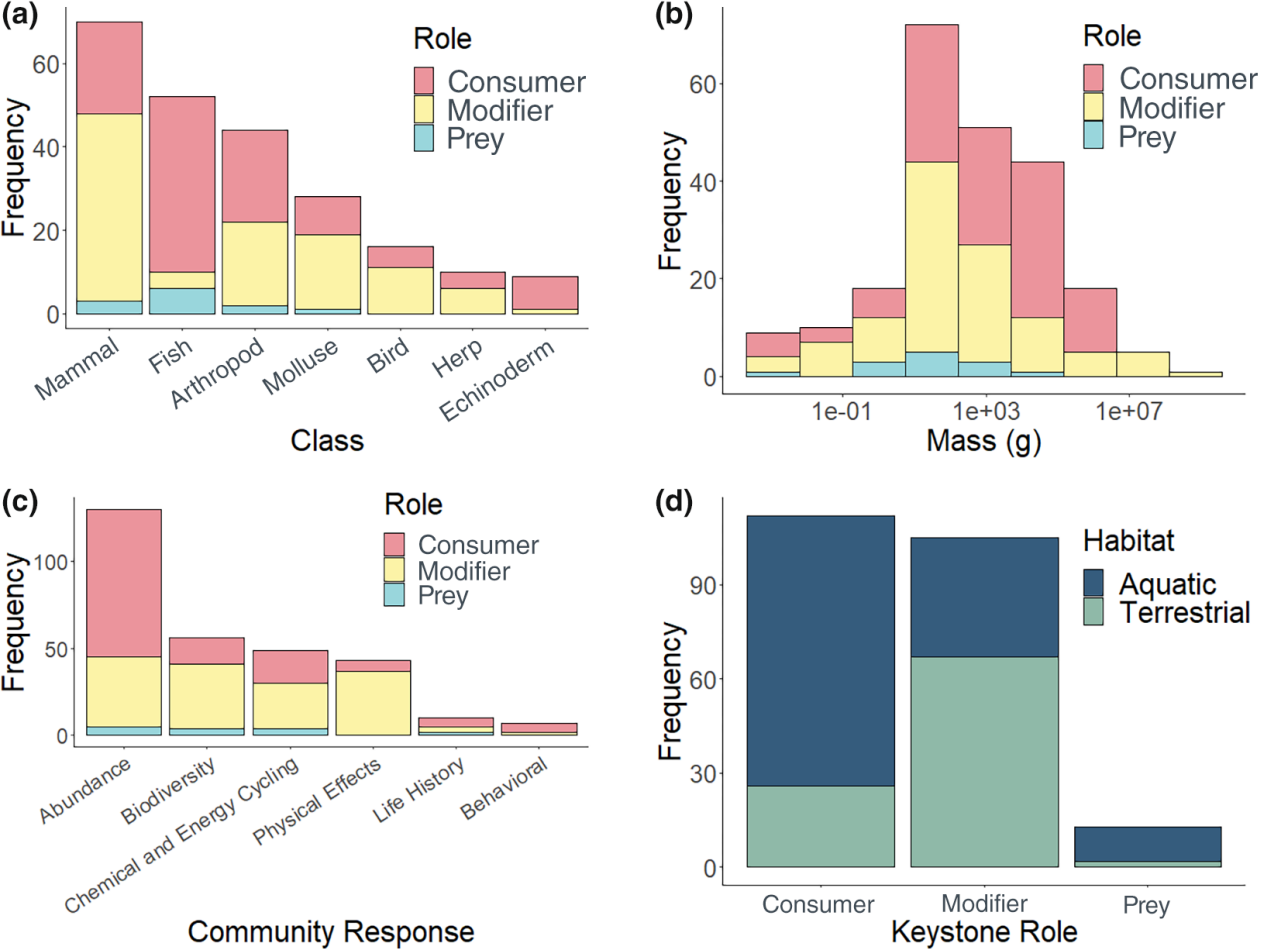
Distribution of keystone traits and their effects on communities and ecosystems. (a) Simplified taxonomic class by keystone role; (b) mass (g) by keystone role; (c) community response by keystone role; (d) keystone role by habitat.

Our search revealed a large diversity of species identified as keystones invoking varied community responses (Table S1). Data from 157 studies led authors to designate 230 species across 17 taxonomic classes (Figure 3a). The most commonly represented classes were mammals (*n* = 70), fish (*n* = 52), arthropods (*n* = 44), and mollusks (*n* = 28). Consumers were the most common keystone roles (50%), followed by modifiers (44%), and prey (5%). Mass distribution was highly right‐skewed, with a median of 431 g and a mean of 1862 kgs (Figure 3b). A change in the abundance of other species was the most common community response (43%), followed by biodiversity changes (19%), chemical and energy cycling (16%), changes to the physical environment (14%), changes in life history (3%), and behavioral changes (2%). Most species (70%) were associated with one community response (Figure 3c). Of the species associated with two measures, the most common additional community responses were changes in biodiversity and chemical and energy cycling (*n* = 23 and 21 species, respectively).

**FIGURE 4:**
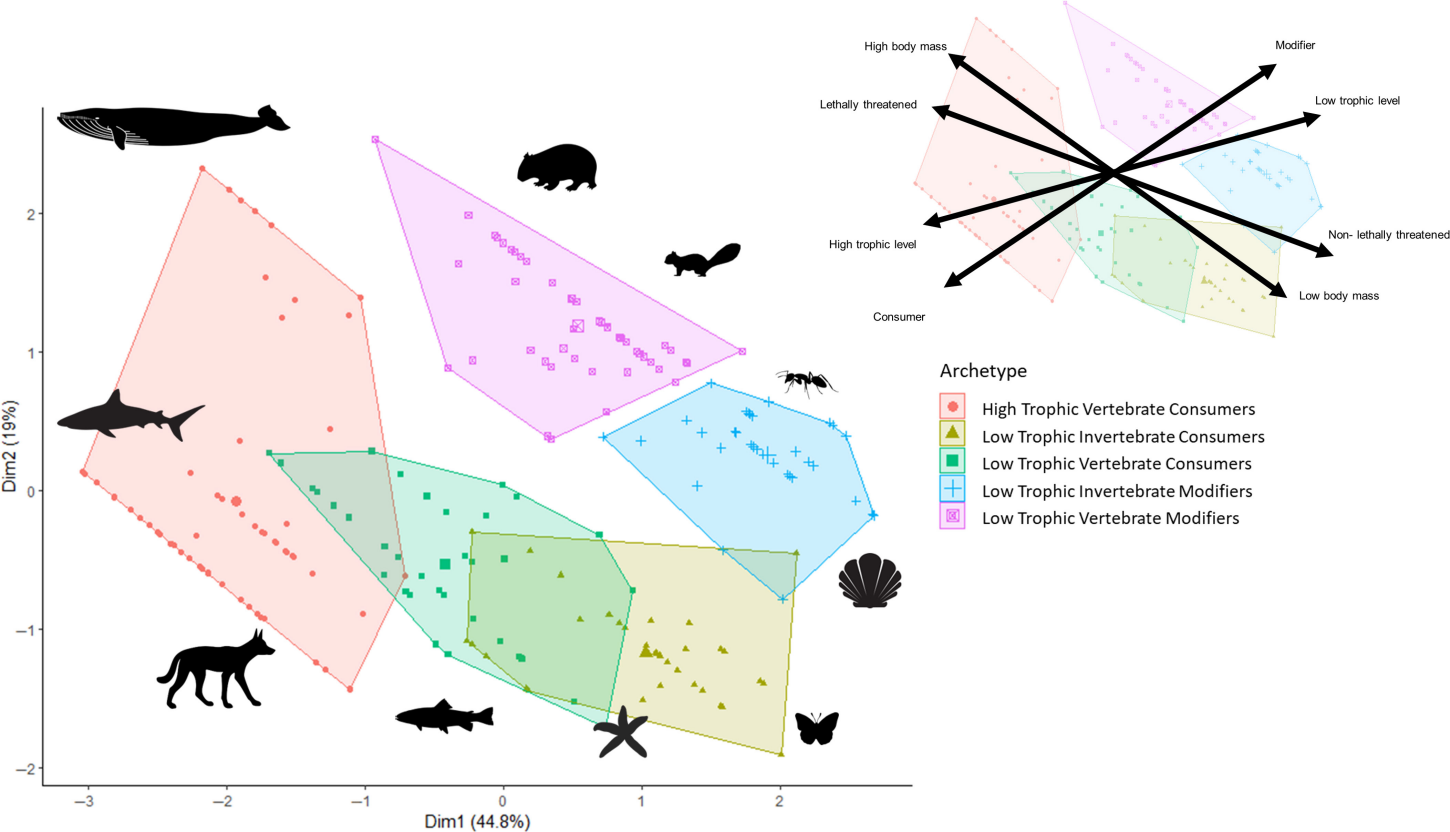
Five keystone archetypes as identified by a k‐means clustering analysis. Axes are pictured top right.

Our clustering analysis revealed five distinct archetypes based on dominant traits of species and the community and ecosystem responses they elicited (Figure 4 and Table 1). Cluster 1 consisted of large‐bodied, high‐trophic‐level vertebrate consumers (e.g., Bull shark, *Carcharhinus leucas*). These high‐trophic‐level vertebrate consumers were likely to elicit trophic cascades; for example, wolves, *Canis lupus*, prey on moose, *Alces alces*, which can influence the growth of Balsam fir, *Abies balsam*, trees (McLaren & Peterson, 1994). Cluster 2 consisted of smaller, lower‐level invertebrate consumers (e.g., Long‐spined Sea urchin, *Diadema africanum*; cabbage butterfly, *Pieris rapae*, etc.). These invertebrate consumers were primarily herbivores that altered vegetation abundance or composition; for example, freshwater pearl mussel, *Margaritifera margaritifera*, fed on macrophytic plants, which can increase water clarity (Geist, 2010). Cluster 3 was dominated by low‐trophic‐level vertebrate consumers (e.g., European sprat, *Sprattus sprattus*; sheepshead bream, *Diplodus puntazzo*, etc.). Similar to cluster 1, this group of mostly fish prey upon smaller invertebrates or detritivores; for example, European bullhead, *Cottus gobiio*, can decrease the abundance of detritivorous freshwater shrimp, *Gammarus pulex*, which can influence decomposition rates (Woodward et al., 2008). Cluster 4 consisted primarily of low‐trophic‐level invertebrate modifiers (e.g., Northern shrimp, *Pandalus borealis*; common cockle, *Cerastoderma edule*, etc.). This cluster included primarily small invertebrates performing ecosystem services; for example, the Western Honey Bee, *Apis mellifera*, can increase the genetic diversity of a number of plants (Traveset et al., 2017). Finally, Cluster 5 was comprised primarily of low‐trophic‐level vertebrate modifiers (e.g., Greater bilby, *Macrotis lagotis*; ice rat, *Otomys sloggetti*, etc.). This cluster included primarily small mammals that performed some level of bioturbation; for example, the Black‐tailed prairie dog, *Cynomys ludovicianus*, that disturbs soils and sediment, and can thereby alter the vegetative community (Duchardt et al., 2021). Clusters 1–3 were mostly aquatic species, whereas 4 and 5 were primarily terrestrial (Table 1).

**TABLE 1:** Descriptive statistics of the five keystone archetypes identified in the cluster analysis. Only elements comprising over 10% are listed.

	Taxonomic class	Average mass	Trophic level	Keystone role	Habitat
Cluster 1 *N* = 60	54% fish 37% mammal	36.3 kg	98% high	78% consumer	76% aquatic
Cluster 2 *N* = 35	61% arthropods 20% mollusk 20% echinoderms	22 g	80% low	97% consumer	78% aquatic
Cluster 3 *N* = 37	66% fish 13% mammal 13% bird	416 g	86% low	89% consumer	71% aquatic
Cluster 4 *N* = 40	47% arthropod 10% mollusk 25% mammals	18 g	97% low	95% modifier	63% terrestrial
Cluster 5 *N* = 44	68% mammal	977 g	90% low	95% modifier	85% terrestrial

## 4 DISCUSSION

Our analysis revealed a wide range of taxonomies, roles, and community responses among species designated as keystones in the literature. Although considerable scientific and popular attention has focused on large terrestrial carnivores, the original keystone predator was a medium‐sized marine invertebrate (Paine, 1966, 1969), and recent research places an emphasis on keystone roles that are equally, if not more, influential than predation (Brock & Kelt, 2004; Brown & Heske, 1990; Davidson et al., 2012). Indeed, the subsequent literature identified many more small‐bodied, low‐trophic‐level keystone species, ranging from cabbage butterfly (*Pieris rapae*) to Mole salamander (*Ambystoma talpoideum*). Current cultural perception surrounding large carnivores might inflate the prominence of their ecosystem effects, thereby discounting smaller‐bodied keystones, or those that affect ecosystems through non‐trophic effects, like modifying substrates, recycling nutrients, and creating habitat. For instance, the numerous studies on the Yellowstone wolves often discount the ecosystem effects of another modifier keystone, the North American beaver, *Castor canadensis*, which might have larger and more direct impacts on river ecology (Gable et al., 2020). Although consumer effects primarily elicited changes in abundance, modifier effects were far more varied (Figure 3c). Modifiers also performed multiple different ecosystem services, including energy and chemical cycling, and habitat creation, as well as affected changes in abundance (Cully Jr et al., 2010; Popescu & Gibbs, 2009; Sutherland & Hill, 1995). Additionally, most keystone consumers identified here occurred in aquatic habitats and were much more likely to be fishes (Figure 3d). Finally, most mammals identified as keystones were not consumers, but modifiers. These were primarily smaller rodents, like the Burrowing bettong (*Bettongia lesueur*), which serves as a bioturbator, altering local vegetative community composition and geochemical cycles (Davidson et al., 2012). Although a list might suggest otherwise, keystone status should not be considered a binary designation for a species. Our list, based on 53 years of literature since the term was coined, reflected large variation in the quality and abundance of evidence for keystone justification. Similarly, our search was limited to keystone animals, excluding possible keystone plants, algae, viruses, bacteria, or other non‐animal keystones. Although these keystones are comparatively rarer in the literature, burgeoning work suggests that these too can have disproportionately large roles in ecosystem function (e.g., Davic, 2003; Power et al., 1985). Our objective was neither to assess the validity of the assertions of keystone species designation nor to evaluate the data quality or claims regarding interaction strength, but rather to provide insight into and summary of the existing keystone literature. Moreover, we note that the working definitions of keystone species have imprecise criteria. Only a small number of definitions provide a clear threshold for data quality (e.g., how large a ‘disproportionately large effect’ must be, or a quantitative ratio of ‘community impacts’ to ‘relative abundance’; Power et al., 1996). Of the definitions that do provide a quantitative metric, very few subsequent keystone studies report the data required (Mills et al., 1993; Power et al., 1996). Furthermore, varying difficulties in recording directly observable community responses (e.g., abundance, biodiversity, etc.) versus harder‐to‐measure indirect community responses (e.g., behavioral changes) could have resulted in an over‐representation of direct community responses (Werner & Peacor, 2003; Wilson et al., 2020). However, the small number of scientists, each with their own research priorities, can only study and evaluate the ‘disproportionate’ effect on only a fraction of other species and responses. Although an association between a species' presence or abundance and community responses does not necessarily identify or imply a mechanism, we depended on study authors to justify keystone status by drawing on their statistical analyses that accounted for other possible correlates, including justification of what constituted ‘disproportionately large effects’. We acknowledge that our work could reinforce the biases of the literature by limiting the definition and studies to papers that allude to the keystone concept, rather than the actual mechanisms that could elicit disproportionate community responses. In reality, a myriad of other species could be identified as keystone in the current literature if the focus was instead restricted to the relative size of responses elicited by changes in the abundance or loss of species. Indeed, much of the Conservation Biology and Ecology literature likely presents data on keystone species that are not labeled as such. Further analysis is warranted to understand whether species designated as keystones (and others not yet identified) indeed invoke clearly defined keystone effects on ecosystems, and how consistent those effects might be.

Despite this uncertainty, recognizing the large variation in potential keystones and their roles allows for a more comprehensive perspective on how particular species and the processes they influence might be important for conservation. We suggest that keystone identification will continue to be critical in identifying possible routes to the restoration of keystone species and their ecosystem roles (Guernsey et al., 2023; Hale & Koprowski, 2018). Notably, one of the primary ways keystone species were identified was via scenarios following their extirpation or decline, and subsequent observation of how the community responded. This identification‐through‐loss paradigm makes the conservation of existing keystones—those known and, perhaps especially, those unknown—of critical importance.

Our list of keystones differs from those typically targeted for conservation. Most species that receive conservation funding are primarily large, charismatic vertebrates (Albert et al., 2018), yet our analysis revealed that large vertebrates represent only a modest proportion of identified animal keystones. This pattern occurred despite potential taxonomic biases in the literature, with many classes such as *Mammalia* and *Actinopterygii* appearing frequently, as opposed to relatively few studies focusing on others, such as *Gastropoda*. Most on our list are smaller‐bodied and comparatively less charismatic, predisposing them to less conservation funding (Donkersley et al., 2022; Muñoz, 2007). Accordingly, a new era of quests for keystones can endeavor to identify these important species before additional losses accrue.

At the intersection of the keystone species concept and human impacts is the hypothesis that humans could function as ‘hyperkeystone’ species (Worm & Paine, 2016). Indeed, the associated hypotheses that humans ultimately elicit large community impacts via endangering keystone species, and can do so via lethal (i.e., exploitation) and non‐lethal (e.g., habitat destruction) processes, requires more detailed consideration. Ideally, the list of putative species compiled here can aid in guiding such further research.

## AUTHOR CONTRIBUTIONS

**Ishana Shukla:** Conceptualization (equal); data curation (lead); formal analysis (lead); investigation (lead); methodology (lead); validation (lead); visualization (lead); writing – original draft (lead); writing – review and editing (lead). **Kaitlyn M. Gaynor:** Methodology (equal); supervision (equal); writing – review and editing (equal). **Boris Worm:** Conceptualization (equal); supervision (equal); writing – review and editing (equal). **Chris T. Darimont:** Conceptualization (equal); funding acquisition (lead); methodology (supporting); project administration (equal); supervision (lead); writing – original draft (supporting); writing – review and editing (lead).

## Supporting information


Table S1.

## Data Availability

All relevant data are uploaded to GitHub and publicly accessible via the following URL: https://github.com/ishana‐s/Keystone_Diversity_Supplemental.
