# Supplementary material for: The diversity of animals identified as keystone species
Source: Ecol Evol. 2023 Oct 9;13(10):e10561. doi: 10.1002/ece3.10561 (PMC10560868; doi:10.1002/ece3.10561)
Supplement: Supplementary file 1 — Table S1. [file ECE3-13-e10561-s001.pdf]

Table S1.
Descriptions of the animals identified as keystone species included in our study, along with the citation, taxonomic Order, community response, and authors' justification for keystoneness.

**Latin name**	**Common name**	**Order**	**Title**	**Authors**	**Year**	**Journal**	**Community response**	**Additional community response**	**Justification for keystoneness**	**Detailed justification**
Acanthaster cf. solaris	Crown-of-Thorns-seastar	Valvatida	Larval cloning in the crown-of-thorns sea star, a keystone coral predator	Allen et al.	2019	Marine Ecology Progress Series	Abundance	Biodiversity	Post-hoc	Determined from literature assessment
Alligator mississippiensis	American Alligator	Crocodilia	Effect of Red Imported Fire Ant Envenomization on Neonatal American Alligators	Allen et al.	1999	Journal of Herpetology	Physical Effects	No secondary response	Post-hoc	Determined from literature assessment
Alosa pseudoharengus	Alewife	Clupeiformes	Intraspecific variation in a predator affects community structure and cascading trophic interactions	Post et al.	2008	Ecology	Life history	No secondary response	Post-hoc	Determined from literature assessment
Alpheus japonicus	Japanese Snapping Shrimp	Decapoda	Using network analysis to identify keystone species in the food web of Haizhou Bay, China	Wu et al.	2019	Marine and Freshwater Research	Abundance	No secondary response	Primary	network

Ambystoma talpoideum	Mole Salamander	Urodela	Identifying potential keystone species from field data – an example from temporary ponds	Fauth	2002	Ecology Letters	Biodiversity	No secondary response	Primary	Empirical observation or experiment
Ammodytes tobianus	Lesser Sand Eel	Trachiniformes	Seabird-fishery interactions: quantifying the sensitivity of seabirds to reductions in sandeel abundance, and identification of key areas for sensitive seabirds in the North Sea	Furness and Tasker	2000	Marine Ecology	Life history	No secondary response	Primary	Empirical observation or experiment
Ammodytidae	Sand Lace	Trachiniformes	Report on identification of keystone species and processes across regional seas	Frederiksen et al.	2006	Journal of Animal Ecology	Life history	No secondary response	Post-hoc	Determined from literature assessment
Anax junius	Common Green Darner	Odonata	Ontogenetic functional diversity: Size structure of a keystone predator drives functioning of a complex ecosystem	Rudolf and Rasmussen	2013	Ecology	Abundance	No secondary response	Primary	Empirical observation or experiment
Anser caerulescens	Snow goose	Anseriformes	Destruction of Wetland Habitats by Lesser Snow Geese: A Keystone Species on the West Coast of Hudson Bay	Kerbes et al.	1990	Journal of Applied Ecology	Biodiversity	No secondary response	Primary	Empirical observation or experiment

Aphaenogaster rudis	Funnel Ant	Hymenoptera	Forest invader replaces predation but not dispersal services by a keystone species	Warren et al.	2015	Biological Invasions	Abundance	No secondary response	Primary	Empirical observation or experiment
Apis mellifera	Western Honey Bee	Hymenoptera	Plant survival and keystone pollinator species in stochastic coextinction models: role of intrinsic dependence on animal-pollination	Traveset et al.	2017	Scientific Reports	Life history	No secondary response	Primary	network
Arbacia lixula	Black Sea Urchin	Arbacioida	Enjoying the warming Mediterranean: Transcriptomic responses to temperature changes of a thermophilous keystone species in benthic communities	Pérez-Portela et al.	2020	Molecular Ecology	Abundance	No secondary response	Post-hoc	Determined from literature assessment
Arenicola marina	Lugworm	Patellogastropoda	Ecosystem engineering in intertidal sand by the lugworm Arenicola marina	Volkenborn	2006	PhD Thesis, University of Bremen	Chemical and Energy Cycling	No secondary response	Post-hoc	Determined from literature assessment
Astacus astacus	Noble Crayfish	Decapoda	Native and alien crayfish species: do their trophic roles differ?	Weinländer and Füreder	2016	Freshwater Science	Chemical and Energy Cycling	No secondary response	Primary	Empirical observation or experiment
Asterias rubens	Common Starfish	Forcipulatida	The stars are out: Predicting the effect of seawater freshening on the ecological impact	Dickey et al.	2021	Ecological Indicators	Abundance	No secondary response	Post-hoc	Determined from literature assessment

			of a sea star keystone predator							
Austropotamobius torrentium	Stone Crayfish	Decapoda	Native and alien crayfish species: do their trophic roles differ?	Weinländer and Füreder	2016	Freshwater Science	Chemical and Energy Cycling	No secondary response	Primary	Empirical observation or experiment
Azteca sericeasur	Arboreal Nesting Ants	Hymenoptera	A Keystone Ant Species Provides Robust Biological Control of the Coffee Berry Borer Under Varying Pest Densities	Morris et al.	2015	PLOS ONE	Abundance	Biodiversity	Post-hoc	Determined from literature assessment
Balaenoptera acutorostrata	Minke Whale	Cetartiodactyla	Existing biodiversity, non-indigenious species, food-web and seafloor integrity GEnS indicators. DEVOTES FP7 Project	Teixeira et al.	2014	NA	Abundance	No secondary response	Post-hoc	Determined from literature assessment
Balaenoptera musculus	Blue Whale	Cetartiodactyla	Southern Ocean iron fertilization by baleen whales and Antarctic krill	Nicol et al.	2010	Fish and Fisheries	Chemical and Energy Cycling	No secondary response	Primary	Empirical observation or experiment
Balaenoptera musculus brevicauda	Pygmy Whale	Cetartiodactyla	Southern Ocean iron fertilization by baleen whales and Antarctic krill	Nicol et al.	2010	Fish and Fisheries	Chemical and Energy Cycling	No secondary response	Primary	Empirical observation or experiment
Balaenoptera physalus	Fin Whale	Cetartiodactyla	Southern Ocean iron fertilization by baleen whales and Antarctic krill	Nicol et al.	2010	Fish and Fisheries	Chemical and Energy Cycling	No secondary response	Primary	Empirical observation or experiment

Balistapus undulatus	Red-lined triggerfish	Tetraodontiformes	Recovery of a coral reef keystone predator, Balistapus undulatus, in East African marine parks	McClanahan	2000	Biological Conservation	Abundance	No secondary response	Primary	Empirical observation or experiment
Bettongia lesueur	Burrowing bettong	Rodentia	Reintroduction of fossorial native mammals and potential impacts on ecosystem processes in an Australian desert landscape	James and Eldridge	2007	Biological Conservation	Chemical and Energy Cycling	No secondary response	Primary	Empirical observation or experiment
Bison bonasus	European bison	Cetartiodactyla	Influence of management practices on large herbivore diet—Case of European bison in Białowieża Primeval Forest (Poland)	Kowalcyzk et al.	2010	Forest Ecology and Management	Abundance	No secondary response	Primary	Empirical observation or experiment
Boreogadus saida	Polar Cod	Gadiformes	Shipping alters the movement and behavior of Arctic cod (Boreogadus saida), a keystone fish in Arctic marine ecosystems	Ivanova et al.	2020	Ecological Applications	Abundance	No secondary response	Post-hoc	Determined from literature assessment
Branchinecta orientalis	Fairy Shrimp	Decapoda	The keystone role of anostracans and copepods in European soda pans during the	Horváth et al.	2013	Freshwater Biology	Biodiversity	No secondary response	Primary	Empirical observation or experiment

			spring migration of waterbirds							
Callianassa filholi	Ghost Shrimp	Decapoda	Temporal and spatial variation in macrofauna community composition imposed by ghost shrimp Callianassa filholi bioturbation	Berkenbusch et al. 2000	2000	Marine Ecology Progress Series	Chemical and Energy Cycling	No secondary response	Primary	Empirical observation or experiment
Canis lupus	Gray Wolf	Carnivora	Trophic cascades linking wolves (Canis lupus), coyotes (Canis latrans), and small mammals	Miller et al.	2012	Canadian Journal of Zoology	Abundance	Behavioral	Primary	Empirical observation or experiment
Canis lupus dingo	Dingo	Carnivora	Keystone effects of an alien top-predator stem extinctions of native mammals	Letnic et al.	2009	Proceedings of the Royal Society B: Biological Sciences	Abundance	No secondary response	Primary	Empirical observation or experiment
Carcharhinus leucas	Bull Shark	Carcharhiniformes	Cascading Effects of the Loss of Apex Predatory Sharks from a Coastal Ocean	Myers et al.	2007	Science	Abundance	No secondary response	Post-hoc	meta-anlsysis
Carcharhinus limbatus	Blacktip Shark	Carcharhiniformes	Cascading Effects of the Loss of Apex Predatory Sharks from a Coastal Ocean	Myers et al.	2007	Science	Abundance	No secondary response	Post-hoc	meta-anlsysis
Carcharhinus obscurus	Dusky Shark	Carcharhiniformes	Cascading Effects of the Loss of Apex Predatory Sharks from a Coastal Ocean	Myers et al.	2007	Science	Abundance	No secondary response	Post-hoc	meta-anlsysis
Carcharhinus plumbeus	Sandbar Shark	Carcharhiniformes	Cascading Effects of the Loss of Apex Predatory	Myers et al.	2007	Science	Abundance	No secondary response	Post-hoc	meta-anlsysis

			Sharks from a Coastal Ocean							
Caretta caretta	Loggerhead Sea Turtle	Testudines	Existing biodiversity, non-indigenious species, food-web and seafloor integrity GEnS indicators. DEVOTES FP7 Project	Teixeira et al.	2014	NA	Abundance	No secondary response	Post-hoc	Determined from literature assessment
Carollia perspicillata	Seba's short tailed bat	Chiroptera	Keystone species in seed dispersal networks are mainly determined by dietary specialization	Mello et al.	2015	Oikos	Abundance	No secondary response	Primary	network
Castor canadensis	North American beaver	Rodentia	Trends in Rocky Mountain amphibians and the role of beaver as a keystone species	Hossack et al.	2015	Biological Conservation	Abundance	Physical Effects	Primary	Empirical observation or experiment
Castor fiber	Eurasian Beaver	Rodentia	Are beavers a solution to the freshwater biodiversity crisis?	Law et al.	2019	Diversity and Distributions	Biodiversity	Physical Effects	Primary	Empirical observation or experiment
Casuarius bennetti	Dwarf Cassowary	Casuariiformes	The Frugivore Community and the Fruiting Plant Flora in a New Guinea Rainforest: Identifying Keystone Frugivores	Mack and Wright	2005	Tropical Fruits and Frugivores	Biodiversity	No secondary response	Primary	network

Centrophorus granulosus	Gulper shark	Squaliformes	The keystone species in the demersal community from the santa maria di leuca cold-water province (mediterranean sea)	Carlucci et al.	2013	Biologia marina mediterranea	Abundance	No secondary response	Primary	network
Cerastoderma edule	Common Cockle	Cardiida	Co-occurrence of pathogen assemblages in a keystone species the common cockle Cerastoderma edule on the Irish coast	Albuixech-Martí et al.	2021	Parasitology	Chemical and Energy Cycling	No secondary response	Post-hoc	Determined from literature assessment
Ceratogymna atrata	Black-casqued Hornbill	Bucerotiformes	Habitat use and resource tracking by African Ceratogymna hornbills: implications for seed dispersal and forest conservation	Whitney and Smith	1998	Animal Conservation	Abundance	Biodiversity	Primary	Empirical observation or experiment
Ceratogymna cylindricus albotibialis	Brown-cheeked Hornbill	Bucerotiformes	Habitat use and resource tracking by African Ceratogymna hornbills: implications for seed dispersal and forest conservation	Whitney and Smith	1998	Animal Conservation	Abundance	Biodiversity	Primary	Empirical observation or experiment
Chaetodipus baileyi	Bailey's Pocket Mouse	Rodentia	Delayed Compensation for Missing Keystone	Ernest and brown	2001	Science	Chemical and Energy Cycling	No secondary response	Primary	Empirical observation or experiment

			Species by Colonization							
Chamelea gallina	Striped Venus	Venerida	Bulgarian Initial assessment and GES Report.	BSBD	2013	NA	Abundance	No secondary response	Post-hoc	Determined from literature assessment
Clupea harengus	Atlantic Herring	Clupeiformes	Human-induced Trophic Cascades and Ecological Regime Shifts in the Baltic Sea	Österblom et al.	2007	Ecosystems	Biodiversity	Chemical and Energy Cycling	Post-hoc	Determined from literature assessment
Colaptes auratus	Yellow-shafted Flicker	Piciformes	Nest sites and nest webs for cavity-nesting communities in interior british columbia, canada: nest characteristics and niche partitioning	Martin et al.	2004	The Condor	Physical Effects	No secondary response	Primary	Empirical observation or experiment
Concholepas concholepas	Chilean abalone	Neogastropoda	Coastal marine communities: trends and perspectives from human-exclusion experiments	Castilla	1999	Trends in Ecology & Evolution	Abundance	No secondary response	Post-hoc	Determined from literature assessment
Connochaetes taurinus	Wildebeest	Artiodactyla	Mammal population regulation, keystone processes and ecosystem dynamics.	Sinclair	2003	Philosophical Transactions of the Royal Society B: Biological Sciences	Abundance	Physical Effects	Primary	network
Cornitermes cumulans	Kollar termite	Blattodea	The Termitaria of Cornitermes cumulans (Isoptera, Termitidae) and Their Role in Determining a	Redford	1984	Biotropica	Biodiversity	No secondary response	Primary	Empirical observation or experiment

			Potential Keystone Species							
Cottus gobio	European Bullhead	Scorpaeniformes	Trophic trickles and cascades in a complex food web: impacts of a keystone predator on stream community structure and ecosystem processes	Woodward et al.	2008	Oikos	Abundance	Chemical and Energy Cycling	Primary	Empirical observation or experiment
Crangon crangon	Brown shrimp	Decapoda	Ecological perspectives of The north sea c. Crangon fishery	Doeksen	2006	BSc Thesis, Wageningen University	Abundance	No secondary response	Post-hoc	Determined from literature assessment
Crocodylus niloticus	Nile crocodile	Crocodilia	Conservation and management of crocodiles in Africa	A.C. Pooley	1973	Journal of the South African Wildlife Management Association	Physical Effects	No secondary response	Post-hoc	Determined from literature assessment
Culter alburnus	Topmouth Cutler	Cypriniformes	Using a topological approach to identify keystone species of fish in eutrophic lake ecosystems: A case of Zhushan Bay, Taihu Lake	Ren et al.	2022	Fisheries Management and Ecology	Abundance	No secondary response	Primary	network
Culter mongolicus	Mongolian Redfin	Cypriniformes	Using a topological approach to identify keystone species of fish in eutrophic lake ecosystems: A case of Zhushan Bay, Taihu Lake	Ren et al.	2022	Fisheries Management and Ecology	Abundance	No secondary response	Primary	network

Cynomys gunnisoni	Gunnison's Prairie Dog	Rodentia	Ecological roles and conservation challenges of social, burrowing, herbivorous mammals in the world's grasslands	Davidson et al.	2013	Frontiers in Ecology and the Environment	Physical Effects	No secondary response	Post-hoc	Determined from literature assessment
Cynomys ludovicianus	Black-tailed Prarie Dog	Rodentia	Direct and indirect effects of a keystone engineer on a shrubland-prairie food web	Duchardt. Et al	2021	Ecology	Biodiversity	Physical Effects	Primary	Empirical observation or experiment
Daphnia galeata	Daphnia spp.	Anomopoda	Daphnia as keystone predators: effects on phytoplankton diversity and grazing resistance	Sarnelle	2005	Journal of Plankton Research	Biodiversity	No secondary response	Primary	Empirical observation or experiment
Daphnia magna	Daphnia spp.	Anomopoda	Daphnia as keystone predators: effects on phytoplankton diversity and grazing resistance	Sarnelle	2005	Journal of Plankton Research	Biodiversity	No secondary response	Primary	Empirical observation or experiment
Dascyllus trimaculatus	Three Spotted Damselfish	Perciformes	The Role of threespot damselfish as a kesytone species in a bahamian patch reef	Axline-Minotti	2003	Msc. Diss. Ohio State University	Behavioral	Biodiversity	Primary	Empirical observation or experiment
Delphinus delphis	Common Dolphin	Cetartiodactyla	Overfishing of top predators eroded the resilience of the Black Sea system regardless of the climate and anthropogenic conditions	Llope et al.	2011	Global Change Biology	Abundance	Chemical and Energy Cycling	Post-hoc	Determined from literature assessment

Delphinus delphis ponticus	Black Sea Common Dolphin	Cetartiodactyla	Overfishing of top predators eroded the resilience of the Black Sea system regardless of the climate and anthropogenic conditions	Llope et al.	2011	Global Change Biology	Abundance	Chemical and Energy Cycling	Post-hoc	Determined from literature assessment
Diadema africanum	Long-spined Sea Urchin	Diadematoida	Before and after a disease outbreak: Tracking a keystone species recovery from a mass mortality event	Gizzi et al.	2020	Marine Environmenta l Research	Abundance	No secondary response	Post-hoc	Determined from literature assessment
Diplodus puntazzo	Sheepshead Bream	Perciformes	Contributions of food web modelling to the ecosystem approach to marine resource management in the Mediterranean Sea	Coll and Libralato	2012	Fish and Fisheries	Abundance	Chemical and Energy Cycling	Primary	network
Dipodomys ingens	Giant Kangaroo Rat	Rodentia	Evaluating current and future range limits of an endangered, keystone rodent (Dipodomys ingens)	Widick and Bean	2019	Diversity and Distributions	Physical Effects	No secondary response	Post-hoc	Determined from literature assessment
Dipodomys merriami	Merriam's Kangaroo Rat	Rodentia	Control of a Desert-Grassland Transition by a Keystone Rodent Guild	Brown and Heske	1990	Science	Abundance	Chemical and Energy Cycling	Primary	Empirical observation or experiment
Dipodomys ordii	Ord's Kangaroo Rat	Rodentia	Control of a Desert-Grassland Transition by a	Brown and Heske	1990	Science	Abundance	Chemical and Energy Cycling	Primary	Empirical observation or experiment

			Keystone Rodent Guild							
Dipodomys spectabilis	Banner-tailed kangaroo rat	Rodentia	Presence/absence of a keystone species as an indicator of rangeland health	Krogh et al	2002	Journal of Arid Environments	Abundance	Chemical and Energy Cycling	Primary	Empirical observation or experiment
Dipodomys stephensi	Stephen's Kangaroo Rat	Rodentia	Keystone effects of the endangered Stephens' kangaroo rat (Dipodomys stephensi)	Brock and Kelt	2004	Biological Conservation	Abundance	Biodiversity	Primary	Empirical observation or experiment
Dobsonia magna	Flying Fox	Chiroptera	The Frugivore Community and the Fruiting Plant Flora in a New Guinea Rainforest: Identifying Keystone Frugivores	Mack and Wright	2005	Tropical Fruits and Frugivores	Abundance	No secondary response	Primary	network
Dolichotis patagonum	Patagonian Mara	Rodentia	Ecological roles and conservation challenges of social, burrowing, herbivorous mammals in the world's grasslands	Davidson et al.	2012	Frontiers in Ecology and the Environment	Physical Effects	No secondary response	Post-hoc	Determined from literature assessment
Donacilla cornea	Donacilla cornea		Bulgarian Initial assessment and GES Report.	BSBD	2013	NA	Abundance	No secondary response	Post-hoc	Determined from literature assessment
Donax trunculus	Abrupt wedge shell	Cardiida	Bulgarian Initial assessment and GES Report.	BSBD	2013	NA	Abundance	No secondary response	Post-hoc	Determined from literature assessment
Dromiciops gliroides	Monito del Monte	Microbiotheria	The disruption of a keystone interaction erodes pollination and	Vitali et al.	2022	Ecology	Biodiversity	No secondary response	Primary	network

			seed dispersal networks							
Dryocopus martius	Black woodpecker	Piciformes	Modelling distribution and potential overlap between Boreal Owl Aegolius funereus and Black Woodpecker Dryocopus martius: implications for management and monitoring plans	Brambilla et al.	2013	Bird Conservation International	Physical Effects	No secondary response	Post-hoc	Determined from literature assessment
Ducula zoeae	Fruit Pigeon	Columbiformes	The Frugivore Community and the Fruiting Plant Flora in a New Guinea Rainforest: Identifying Keystone Frugivores	Mack and Wright	2005	Tropical Fruits and Frugivores	Abundance	No secondary response	Primary	network
Echinus esculentus	Edible sea urchin	Camarodonta	Sea urchin grazing and kelp re-vegetation in the NE Atlantic	Norderhaug and Christie	2009	Marine Biology Research	Abundance	No secondary response	Post-hoc	Determined from literature assessment
Eciton burchelli	Army Ant	Hymenoptera	Habitat fragmentation, percolation theory and the conservation of a keystone species	Boswell et al.	1998	Proceedings of the Royal Society of London. Series B: Biological Sciences	Biodiversity	Physical Effects	Primary	network
Engraulis encrasicolus	European Anchovy	Clupeiformes	Overfishing drives a trophic cascade in the Black Sea	Daskalov	2022	Marine Ecology Progress Series	Abundance	No secondary response	Post-hoc	Determined from literature assessment

Enhydra lutris	Sea Otter	Carnivora	Limited effects of a keystone species:trends of sea otters and kelp forests at the Semichi Islands, Alaska	Konar	2000	Marine Ecology Progress Series	Abundance	Life History	Primary	Empirical observation or experiment
Epinephelus marginatus	Dusky Grouper	Perciformes	Rapid Decline of Nassau Grouper Spawning Aggregations in Belize: Fishery Management and Conservation Needs	Sala et al.	2001	Fisheries	Abundance	No secondary response	Post-hoc	Determined from literature assessment
Eschrichtius robustus	Gray Whale	Cetartiodactyla	Destruction and Opportunity on the Sea Floor: Effects of Gray Whale Feeding	Oliver and Slattery	1985	Ecology	Biodiversity	Physical Effects	Primary	Empirical observation or experiment
Esox lucius	Northern Pike	Esociformes	Salmonid or nonsalmonid lakes: predicting the fate of northern boreal fish communities with hierarchical filters relating to a keystone piscivore	Spens and Ball	2008	Canadian Journal of Fisheries and Aquatic Sciences	Chemical and Energy Cycling	No secondary response	Primary	Empirical observation or experiment
Euastacas armatus	Murray Crayfish	Decapoda	It’s not there, but it could be: a renewed case for reintroduction of a keystone species into the Lower River Murray	Whiterod and Zukowski	2019	Transactions of the Royal Society of South Australia	Chemical and Energy Cycling	No secondary response	Primary	Empirical observation or experiment

Euhrychiopsis lecont	Weevil	Coleoptera	Is There a New Keystone Species in North American Lakes and Rivers?	Creed	2000	Oikos	Abundance	No secondary response	Post-hoc	Determined from literature assessment
Eupomacentrus planifrons	Threespot Damselfish	Perciformes	The Threespot Damselfish: A noncarnivorous keystone species	Williams	1990	The American Natutalist	Behavioral	Biodiversity	Post-hoc	Determined from literature assessment
Eurosta solidaginis	Goldenrod gall Fly	Diptera	Keystone Individuals Alter Ecological and Evolutionary Consumer-Resource Dynamics	Start	2018	The American Naturalist	Life history	No secondary response	Primary	Empirical observation or experiment
Faxonius rusticus	Rusty Crayfish	Decapoda	How a trematode parasite (Microphallus Ward, 1901) impacts the grazing behavior of an aquatic keystone species, the rusty crayfish Faxonius rusticus Girard, 1852 (Decapoda: Decapoda: Astacidea: Cambaridae)	MacKay and Moore	2021	Journal of Crustacean Biology	Abundance	No secondary response	Post-hoc	Determined from literature assessment
Formica rufa	Red Wood Ant	Hymenoptera	The distribution of a group of keystone species is not associated with anthropogenic habitat disturbance	Fitzpatrick et al.	2021	Diversity and Distributions	Abundance	Biodiversity	Primary	network

Gadus morhua	Atlantic Cod	Gadiformes	Meta-Analysis of Cod–Shrimp Interactions Reveals Top-down Control in Oceanic Food Webs	Worm and Meyers	2003	Ecology	Abundance	No secondary response	Post-hoc	meta-anlsysis
Galeocerdo cuvier	Tiger Shark	Carcharhiniformes	Patterns of top-down control in a seagrass ecosystem: could a roving apex predator induce a behaviour-mediated trophic cascade?	Burkholder et al	2012	Journal of Animal Ecology	Behavioral	Life History	Primary	Empirical observation or experiment
Galeus melastomus	Blackmouth Catshark	Carcharhiniformes	The keystone species in the demersal community from the santa maria di leuca cold-water province (mediterranean sea)	Carlucci et al.	2013	Biologia marina mediterranea	Abundance	No secondary response	Primary	network
Gammarus fossarum	Freshwater Shrimp	Decapoda	Additive effects of temperature and infection with an acanthocephalan parasite on the shredding activity of Gammarus fossarum (Crustacea: Amphipoda): the importance of aggregative behavior	Labaude et al.	2017	Global Change Biology	Chemical and Energy Cycling	No secondary response	Post-hoc	Determined from literature assessment

Gecarcoidea natalis	Christmas Island red crab	Decapoda	Invasional 'meltdown' on an oceanic island	O'Dowd et al.	2003	Ecology Letters	Abundance	Chemical and Energy Cycling	Post-hoc	Determined from literature assessment
Geomys bursarius	Plains Pocket Gopher	Rodentia	Effects of Plains Pocket Gopher (Geomys bursarius) Disturbances on Tallgrass-prairie Plant Community Structure	Rogers et al	2013	The American midland Naturalist	Biodiversity	Physical Effects	Primary	Empirical observation or experiment
Glaucomys sabrinus	Northern Flying Squirrel	Rodentia	Ecology of Glaucomys sabrinus: Habitat, Demography, and Community Relations	Smith et al.	2007	Journal of Mammology	Chemical and Energy Cycling	No secondary response	Post-hoc	meta-anlsysis
Gopherus polyphemus	Gopher tortoise	Testudines	Functional relationships reveal keystone effects of the gopher tortoise on vertebrate diversity in a longleaf pine savanna	Catano and Stout	2015	Biodiversity and Conservation	Biodiversity	Physical Effects	Primary	Empirical observation or experiment
Haematopus moquini	African black oystercatchers	Charadriiformes	Diet of the African black oystercatcher Haematopus moquini on rocky shores: spatial, temporal and sex-related variation	Hockey and Branch	1984	African Zoology	Abundance	No secondary response	Primary	Empirical observation or experiment
Halichoerus grypus	Grey seal	Carnivora	Using sensitivity analysis to identify keystone species and keystone links in	Berg et al.	2011	Oikos	Biodiversity	No secondary response	Primary	network

			size-based food webs							
Hesperoleucus symmetricus	California Roach	Cypriniformes	Floods, Food Chains, and Ecosystem Processes in Rivers	Power	1995	Linking Species and Ecosystems	Abundance	Chemical and Energy Cycling	Post-hoc	Determined from literature assessment
Ips typographus	Spruce Bark Beetle	Coleoptera	The European spruce bark beetle Ips typographus in a national park: from pest to keystone species	Müller et al.	2008	Biodiversity and Conservation	Abundance	Biodiversity	Primary	Empirical observation or experiment
Jasus edwardsii	Southern Rock Lobster	Decapoda	Lobsters as keystone: Only in unfished ecosystems?	Eddy et al.	2014	Ecological Modelling	Chemical and Energy Cycling	No secondary response	Primary	network
Lagostomus maximus	Plains Vizcacha	Lagomorpha	Ecological roles and conservation challenges of social, burrowing, herbivorous mammals in the world's grasslands	Davidson et al.	2012	Frontiers in Ecology and the Environment	Physical Effects	No secondary response	Post-hoc	Determined from literature assessment
Larimichthys polyactis	Redlip Croaker	Acanthuriformes	Using network analysis to identify keystone species in the food web of Haizhou Bay, China	Wu et al.	2019	Marine and Freshwater Research	Abundance	No secondary response	Primary	network
Larus audouinii	Audouin's Gull	Charadriiformes	Contributions of food web modelling to the ecosystem approach to marine resource management in the Mediterranean Sea	Coll and Libralato	2012	Fish and Fisheries	Abundance	No secondary response	Post-hoc	Determined from literature assessment

Lasiorhinus latifrons	Southern hairy-nosed wombat	Diprotodontia	Ecological roles and conservation challenges of social, burrowing, herbivorous mammals in the world's grasslands	Davidson et al.	2012	Frontiers in Ecology and the Environment	Physical Effects	No secondary response	Post-hoc	Determined from literature assessment
Laternula elliptica	Saltwater Clam	Heterodonta	Anatomy and behavior of Laternula elliptica, a keystone species of the Antarctic benthos (Bivalvia: Anomalodesmata: Laternulidae)	Passos et al.	2022	PeerJ	Chemical and Energy Cycling	No secondary response	Post-hoc	Determined from literature assessment
Leander modestus	Chinese white prawn	Decapoda	Selection of keystone species based on stable carbon and nitrogen isotopes to construct a typical food web on the shore of Xingkai Lake, China	Xing et al.	2021	Ecological Indicators	Abundance	No secondary response	Primary	network
Lentidium mediterraneum	Lentidium mediterraneum	Myida	Bulgarian Initial assessment and GES Report.	BSBD	2013	NA	Abundance	No secondary response	Post-hoc	Determined from literature assessment
Leptochela gracilis	Lesser Glass Shrimp	Decapoda	Using network analysis to identify keystone species in the food web of Haizhou Bay, China	Wu et al.	2019	Marine and Freshwater Research	Abundance	No secondary response	Primary	network
Leptuca leptodactyla	Thin-fingered fiddler crab	Decapoda	Mangrove crabs as ecosystem engineers; with	Kristensen	2008	Mangrove Macrobenthos Special Issue	Chemical and Energy Cycling	Physical Effects	Primary	Empirical observation or experiment

			emphasis on sediment processes							
Leptuca uruguayensis	Uruguayan fiddler crab	Decapoda	Mangrove crabs as ecosystem engineers; with emphasis on sediment processes	Kristensen	2008	Mangrove Macrobenthos Special Issue	Chemical and Energy Cycling	Physical Effects	Primary	Empirical observation or experiment
Lepus americanus	Snowshoe hare	Lagomorpha	Prey availability and ambient temperature influence carrion persistence in the boreal forest	Peers et al.	2020	Journal of Animal Ecology	Chemical and Energy Cycling	No secondary response	Primary	Empirical observation or experiment
Limaria hians	Gaping file shell	Limida	Limaria hians (Mollusca: Limacea): a neglected reef-forming keystone species	Hall-Spencer and Moore	2000	Aquatic Conservation: Marine and Freshwater Ecosystems	Physical Effects	Biodiversity	Post-hoc	Determined from literature assessment
Loligo plei	Tropical Arrow squid	Myopsida	The trophic role of the squid Loligo plei as a keystone species in the South Brazil Bight ecosystem	Gasalla et al.	2010	ICES Journal of Marine Science	Abundance	No secondary response	Primary	network
Lophius budegassa	Blackbellied Angler	Lophiiformes	The keystone species in the demersal community from the santa maria di leuca cold-water province (mediterranean sea)	Carlucci et al.	2013	Biologia marina mediterranea	Abundance	No secondary response	Primary	network
Loxodonta africana	African elephant	Proboscidea	Elephants and Fire as Causes of Multiple Stable	Dublin	1990	Journal of Animal Ecology	Abundance	Physical Effects	Primary	Empirical observation or experiment

			States in the Serengeti-Mara Woodlands							
Lynx lynx	Eurasian Lynx	Carnivora	Usability of large carnivore as a keystone species in Eastern Black Sea Region, Turkey	Ucarli	2011	African Journal of Biotechnology	Abundance	No secondary response	Post-hoc	Determined from literature assessment
Lynx pardinus	Iberian lynx	Carnivora	Positive Effects on Game Species of Top Predators by Controlling Smaller Predator Populations: An Example with Lynx, Mongooses, and Rabbits	Palomares et al	1994	Conservation Biology	Abundance	No secondary response	Primary	Empirical observation or experiment
Macoma balthica	Baltic clam	Cardiida	Burrowing behaviour of the Baltic clam Macoma balthica: effects of sediment type, hypoxia and predator presence	Tallqvist	2001	Marine Ecology Progress Series	Chemical and Energy Cycling	No secondary response	Primary	Empirical observation or experiment
Macrotis lagotis	Greater Bilby	Rodentia	Reintroduction of fossorial native mammals and potential impacts on ecosystem processes in an Australian desert landscape	James and Eldridge	2007	Biological Conservation	Biodiversity	Chemical and Energy Cycling	Primary	Empirical observation or experiment
Margaritifera margaritifera	Freshwater Pearl Mussel	Unionida	Climate Warming as a Possible Trigger of Keystone Mussel Population	Bolotov et al.	2018	Scientific Reports	Abundance	No secondary response	Post-hoc	Determined from literature assessment

			Decline in Oligotrophic Rivers at the Continental Scale							
Marmota himalayana	Himalayan Marmot	Rodentia	Ecological roles and conservation challenges of social, burrowing, herbivorous mammals in the world's grasslands	Davidson et al.	2016	Frontiers in Ecology and the Environment	Physical Effects	No secondary response	Post-hoc	Determined from literature assessment
Marmota sibirica	Siberian Marmot	Rodentia	Ecological roles and conservation challenges of social, burrowing, herbivorous mammals in the world's grasslands	Davidson et al.	2015	Frontiers in Ecology and the Environment	Physical Effects	No secondary response	Post-hoc	Determined from literature assessment
Mecistops cataphractus	Slender-snouted crocodile	Crocodilia	Conservation and management of crocodiles in Africa	A.C. Pooley	1973	Journal of the South African Wildlife Management Association	Physical Effects	No secondary response	Post-hoc	Determined from literature assessment
Megaptera novaeangliae	Humpback Whale	Cetartiodactyla	Southern Ocean iron fertilization by baleen whales and Antarctic krill	Nicol et al.	2010	Fish and Fisheries	Chemical and Energy Cycling	No secondary response	Primary	Empirical observation or experiment
Merluccius merluccius	European Hake	Gadiformes	Contributions of food web modelling to the ecosystem approach to marine resource management in the Mediterranean Sea	Coll and Libralato	2012	Fish and Fisheries	Abundance	Chemical and Energy Cycling	Post-hoc	Determined from literature assessment
Meyenaster gelatinosus	Large Spiny Seastar	Forcipulatida	Quantifying keystone species complexes:	Ortiz et al.	2017	Ecological Indicators	Physical Effects	No secondary response	Primary	network

			Ecosystem-based conservation management in the King George Island (Antarctic Peninsula)							
Micropterus punctulatus	Spotted Bass	Perciformes	Grazing Minnows, Piscivorous Bass, and Stream Algae: Dynamics of a Strong Interaction	Power and Gregoire	1978	Ecology	Abundance	No secondary response	Primary	Empirical observation or experiment
Micropterus salmoides	Largemouth Bass	Perciformes	Perturbation and Resilience: A Long-Term, Whole-Lake Study of Predator Extinction and Reintroduction	Mittelbach et al.	1995	Ecology	Abundance	Life History	Primary	Empirical observation or experiment
Microrhopala vittata	Chrysomelid beetle	Coleoptera	Herbivory and Plant Species Coexistence: Community Regulation by an Outbreaking Phytophagous Insect	Carson and Root	2000	Ecological Monographs	Abundance	Biodiversity	Primary	Empirical observation or experiment
Modiolula phaseolina	Bean Horse Mussel	Mytilida	Bulgarian Initial assessment and GES Report.	BSBD	2013	NA	Abundance	No secondary response	Post-hoc	Determined from literature assessment
Modiolus modiolus	Horse mussel	Mytilida	Community convergence and recruitment of keystone species as performance indicators of artificial reefs	Fariñas-Franco et al.	2013	Journal of Sea Research	Physical Effects	No secondary response	Primary	Empirical observation or experiment

Monachus monachus	Mediterranean Monk Seal	Carnivora	Overfishing drives a trophic cascade in the Black Sea	Daskalov	2022	Marine Ecology Progress Series	Abundance	No secondary response	Post-hoc	Determined from literature assessment
Myliobatis californica	Bat Ray	Myliobatiformes	Experimental Analyses of Structural Regulation in a Marine Sand Community Exposed to Oceanic Swell	Van Blaricom	1982	Ecological Monographs	Biodiversity	Physical Effects	Primary	Empirical observation or experiment
Mysis diluviana	Opossum Shrimp	Mysida	Hydroclimate mediates effects of a keystone species in a coldwater reservoir	Johnson and Martinez	2012	Lake and Reservoir Management	Abundance	Chemical and Energy Cycling	Primary	Empirical observation or experiment
Mytilaster lineatus	Mytilaster lineatus	Mytilida	Existing biodiversity, non-indigenious species, food-web and seafloor integrity GEnS indicators. DEVOTES FP7 Project	Teixeira et al.	2014	NA	Abundance	No secondary response	Post-hoc	Determined from literature assessment
Mytilus edulis	Blue Mussel	Mytilida	Effects of cigarette butts on marine keystone species (Ulva lactuca L. and Mytilus edulis L.) and sediment microphytobenthos	Green et al.	2021	Marine Pollution Bulletin	Abundance	Biodiversity	Post-hoc	Determined from literature assessment
Mytilus galloprovincialis	Mediterranean mussel	Mytilida	Existing biodiversity, non-indigenious species, food-web	Teixeira et al.	2014	NA	Abundance	No secondary response	Post-hoc	Determined from literature assessment

			and seafloor integrity GEnS indicators. DEVOTES FP7 Project							
Mytilus galloprovincialis	Mediterraneum Mussel	Mytilida	Bulgarian Initial assessment and GES Report.	BSBD	2013	NA	Abundance	No secondary response	Post-hoc	Determined from literature assessment
Mytilus trossulus	Bay Mussel	Mytilida	Importance of ice algae and pelagic phytoplankton as food sources revealed by fatty acid trophic markers in a keystone species (Mytilus trossulus) from the High Arctic	Thyrring et al.	2017	Marine Ecology Progress Series	Abundance	No secondary response	Post-hoc	Determined from literature assessment
Neotoma albigula	Western White-Throated Woodrat	Rodentia	Pack rats (Neotoma spp.): Keystone ecological engineers?	Whitford and Steinberger	2021	Journal of Arid Environments	Biodiversity	Chemical and Energy Cycling	Primary	Empirical observation or experiment
Neotoma micropus	Southern Plains Woodrat	Rodentia	Pack rats (Neotoma spp.): Keystone ecological engineers?	Whitford and Steinberger	2021	Journal of Arid Environments	Biodiversity	Chemical and Energy Cycling	Primary	Empirical observation or experiment
Nocomis biguttatus	Hornyhead Chub	Cypriniformes	Nesting Ecology and Behavior of Hornyhead Chub Nocomis biguttatus, a Keystone Species in Allequash Creek, Wisconsin	Vives	1990	The American Midland Naturalist	Biodiversity	Physical Effects	Post-hoc	Determined from literature assessment
Notophthalmus viridescens	Eastern Newt	Urodela	Identifying potential keystone species from field	Fauth	2002	Ecology Letters	Biodiversity	No secondary response	Primary	Empirical observation or experiment

			data – an example from temporary ponds							
Ochotona curzoniae	Plateau Pika	Lagomorpha	Keystone status of plateau pikas (Ochotona curzoniae): effect of control on biodiversity of native birds	Lai and Smith	2003	Biodiversity and Conservation	Abundance	Biodiversity	Primary	Empirical observation or experiment
Ochotona princeps	American Pika	Rodentia	Influence of Refuging Consumers (Pikas: Ochotona Princeps) on Subalpine Meadow Vegetation	Huntly	1987	Ecology	Abundance	Biodiversity	Primary	Empirical observation or experiment
Octodon degus	Degu	Rodentia	Ecological roles and conservation challenges of social, burrowing, herbivorous mammals in the world's grasslands	Davidson et al.	2012	Frontiers in Ecology and the Environment	Physical Effects	No secondary response	Post-hoc	Determined from literature assessment
Odocoileus virginianus	White tailed Deer	Artiodactyla	The White-Tailed Deer: A Keystone Herbivore	Waller and Alverson	1997	Wildlife Society Bulletin	Abundance	Biodiversity	Primary	Empirical observation or experiment
Oedemera flavipes	Oedemera flavipes	Coleoptera	Plant survival and keystone pollinator species in stochastic coextinction models: role of intrinsic dependence on animal-pollination	Traveset et al.	2017	Scientific Reports	Biodiversity	No secondary response	Primary	network

Oncorhynchus nerka	Sockeye Salmon	Salmoniformes	Keystone Interactions: Salmon and Bear in Riparian Forests of Alaska	Helfield and Naiman	2006	Ecosystems	Chemical and Energy Cycling	No secondary response	Primary	Empirical observation or experiment
Ophiothrix fragilis	Common Brittlestar	Ophiurida	Near-future level of CO2-driven ocean acidification radically affects larval survival and development in the brittlestar Ophiothrix fragilis	Dupont et al.	2008	Marine Ecology Progress Series	Abundance	Biodiversity	Post-hoc	Determined from literature assessment
Oratosquilla oratoria	Japanese Mantis Shrimp	Stomatopoda	Using network analysis to identify keystone species in the food web of Haizhou Bay, China	Wu et al.	2019	Marine and Freshwater Research	Abundance	No secondary response	Primary	network
Oryctolagus cuniculus	European Rabbit	Lagomorpha	Disease-mediated bottom-up regulation: An emergent virus affects a keystone prey, and alters the dynamics of trophic webs	Monterroso et al.	2016	Scientific Reports	Abundance	No secondary response	Primary	Empirical observation or experiment
Osteolaemus tetraspis	African Dwarf Crocodile	Crocodilia	Conservation and management of crocodiles in Africa	A.C. Pooley	1973	Journal of the South African Wildlife Management Association	Physical Effects	No secondary response	Post-hoc	Determined from literature assessment
Ostrea edulis	European oyster	Ostreida	Conservation and restoration of a keystone species: Understanding the settlement	Rodriguez-Perez et al.	2019	Marine Pollution Bulletin	Physical Effects	No secondary response	Post-hoc	Determined from literature assessment

			preferences of the European oyster (Ostrea edulis)							
Ostrea edulis	Native oyster	Ostreida	The European oyster (Ostrea edulis) and its epibiotic succession	Smyth and Roberts	2010	Hydrobiologia	Biodiversity	No secondary response	Post-hoc	Determined from literature assessment
Otomys sloggetti	Ice Rat	Rodentia	Does the ice rat Otomys sloggetti robertsi contribute to habitat change in Lesotho?	Mokotjomela et al.	2009	Acta Oecologica	Biodiversity	Chemical and Energy Cycling	Primary	Empirical observation or experiment
Otospermophilus beecheyi	California Ground Squirrel	Rodentia	Ecological roles and conservation challenges of social, burrowing, herbivorous mammals in the world's grasslands	Davidson et al.	2012	Frontiers in Ecology and the Environment	Physical Effects	No secondary response	Post-hoc	Determined from literature assessment
Pagophilus groenlandicus	Harp Seal	Carnivora	An Ecopath Model for the Norwegian and Barents Sea.	Dommasnes et al.	2001	NA	Abundance	No secondary response	Post-hoc	Determined from literature assessment
Pandalus borealis	Northern Shrimp	Decapoda	DNA metabarcoding reveals the importance of gelatinous zooplankton in the diet of Pandalus borealis, a keystone species in the Arctic	Urban et al.	2022	Molecular Ecology	Chemical and Energy Cycling	No secondary response	Post-hoc	Determined from literature assessment
Panthera onca	Jaguar	Carnivora	Ecological Meltdown in Predator-Free Forest Fragments	Terborgh et al.	2001	Science	Abundance	Biodiversity	Primary	Empirical observation or experiment

Paracentrotus lividus	Purple Sea Urchin	Camarodonta	East is East and West is West: Population genomics and hierarchical analyses reveal genetic structure and adaptation footprints in the keystone species Paracentrotus lividus (Echinoidea)	Carreras et al.	2020	Diversity and Distributions	Abundance	No secondary response	Post-hoc	Determined from literature assessment
Paralonchurus brasiliensis	Banded Croaker	Perciformes	Reproductive strategy and fecundity of the keystone species Paralonchurus brasiliensis (Teleostei, Sciaenidae): an image processing techniques application	Costa et al.	2015	Environmental Biology of Fishes	Abundance	No secondary response	Post-hoc	Determined from literature assessment
Paranephrops planifrons	Freshwater Crayfish	Decapoda	Koura: a keystone species?	Collier et al.	1997	Stream Ecosystems	Abundance	No secondary response	Primary	Empirical observation or experiment
Parasesarma messa	Maroon Mangrove Crab	Amphionidacea	Keystone species and mangrove forest dynamics: the influence of burrowing by crabs on soil nutrient status and forest productivity	Smith et al.	1991	Estuarine, Coastal and Shelf Science	Chemical and Energy Cycling	No secondary response	Primary	Empirical observation or experiment
Patella aspera	Atlantic Limpet	Patellogastropoda	Growth and reproduction of the north-eastern Atlantic keystone	Sousa et al.	2017	Helgoland Marine Research	Physical Effects	No secondary response	Post-hoc	Determined from literature assessment

			species Patella aspera (Mollusca: Patellogastropoda)							
Patella candei	Sun limpet	Patella candei	Community structure and dynamics of the azorean rocky intertidal: exploitation of keystone species	Martins	2008	PhD Thesis, Univ. of Plymouth	Abundance	No secondary response	Primary	Empirical observation or experiment
Patella rustica	High-shore Limpet	Patellogastropoda	Growth and Morphological Trends of the Keystone Species Patella rustica Linnaeus, 1758 (Mollusca: Gastropoda) in a Protected Mediterranean Lagoon	Bensaâd-Bendjedid et al.	2022	Acta Zoologica Bulgarica	Abundance	No secondary response	Post-hoc	Determined from literature assessment
Patella vulgata	Common Limpet	Patellogastropoda	A continental scale evaluation of the role of limpet grazing on rocky shores	Coleman et al.	2006	Oecologia	Abundance	No secondary response	Post-hoc	Determined from literature assessment
Pedetes capensis	Springhare	Rodentia	Ecological roles and conservation challenges of social, burrowing, herbivorous mammals in the world's grasslands	Davidson et al.	2012	Frontiers in Ecology and the Environment	Physical Effects	No secondary response	Post-hoc	Determined from literature assessment
Perca fluviatilis	European Perch	Perciformes	Fish Predation and Benthic Community Structure: The Role of Omnivory and	Diehl	1992	Ecology	Abundance	Life History	Primary	Empirical observation or experiment

			Habitat Complexity							
Phalacrocorax carbo	Great Cormorant	Suliformes	Food consumption by seabirds in Norwegian waters	Barrett et al.	2002	ICES Journal of Marine Science	Abundance	No secondary response	Primary	network
Phoca vitulina	Harbor Seal	Carnivora	Existing biodiversity, non-indigenious species, food-web and seafloor integrity GEnS indicators. DEVOTES FP7 Project	Teixeira et al.	2014	NA	Abundance	No secondary response	Post-hoc	Determined from literature assessment
Phocoena phocoena	Harbor Porpoise	Cetartiodactyla	Overfishing drives a trophic cascade in the Black Sea	Daskalov	2002	Marine Ecology Progress Series	Abundance	No secondary response	Post-hoc	Determined from literature assessment
Phocoena phocoena relicta	Black Sea Harbor Porpoise	Cetartiodactyla	Overfishing drives a trophic cascade in the Black Sea	Daskalov	2002	Marine Ecology Progress Series	Abundance	No secondary response	Post-hoc	Determined from literature assessment
Pholas dactylus	Common piddock	Myida	Bulgarian Initial assessment and GES Report.	BSBD	2013	NA	Abundance	No secondary response	Post-hoc	Determined from literature assessment
Pieris rapae	Cabbage White Butterfly	Lepidoptera	Herbivore-induced plant responses in Brassica oleracea prevail over effects of constitutive resistance and result in enhanced herbivore attack	Poelman and Kessler	2010	Ecological Entomology	Abundance	No secondary response	Primary	Empirical observation or experiment
Pisaster ochraceus	Purple Sea Star	Forcipulatida	The Pisaster-Tegula Interaction: Prey Patches, Predator	Paine	1969	The American Naturalist	Abundance	Biodiversity	Primary	Empirical observation or experiment

			Food Preference, and Intertidal Community Structure							
Pomatomus saltatrix	Bluefish	Perciformes	Overfishing drives a trophic cascade in the Black Sea	Daskalov	2002	Marine Ecology Progress Series	Abundance	No secondary response	Post-hoc	Determined from literature assessment
Pomatoschistus microps	Common Goby	Gobiiformes	An abundant small sized fish as keystone species? The effect of Pomatoschistus microps on food webs and its trophic role in two intertidal benthic communities: A modeling approach	Pockberger et al.	2014	Journal of Sea Research	Abundance	No secondary response	Primary	network
Pristomyrmex pungens Mayr	Japanese Queenless Ant	Hymenoptera	Leaf Volatiles from Two Corydalis Species Lure A Keystone Seed-dispersing Ant and Enhance Seed Retrieval	Zhu and Wang	2018	Sociobiology	Abundance	Biodiversity	Post-hoc	Determined from literature assessment
Procambarus alleni	Everglages Crayfish	Decapoda	The effect of hydroperiod on the growth of the crayfish species Procambarus alleni and Procambarus fallax: Two keystone species in the Florida Everglades	Gardener	2006	MSc Diss. Florida Atlantic University	Abundance	Chemical and Energy Cycling	Post-hoc	Determined from literature assessment

Procambarus fallax	Deceitful Crayfish	Decapoda	The effect of hydroperiod on the growth of the crayfish species Procambarus alleni and Procambarus fallax: Two keystone species in the Florida Everglades	Gardener	2006	MSc Diss. Florida Atlantic University	Abundance	Chemical and Energy Cycling	Post-hoc	Determined from literature assessment
Pterodroma gouldi	Grey-faced Petrel	Procellariiformes	Monitoring burrowing petrel populations: A sampling scheme for the management of an island keystone species	Buxton et al.	2016	The Journal of Wildlife Management	Abundance	Chemical and Energy Cycling	Primary	Empirical observation or experiment
Pteropus dasymallus inopinatus	Orii's flying fox	Chiroptera	The role of Orii's flying-fox (Pteropus dasymallus inopinatus) as a pollinator and a seed disperser on Okinawa-jima Island, the Ryukyu Archipelago, Japan	Nakamoto et al.	2008	Ecological Research	Abundance	No secondary response	Primary	Empirical observation or experiment
Pteropus samoensis	Samoan Flying Fox	Chiroptera	Topological keystone species in ecological interaction networks: Considering link quality and non-trophic effects	Vasas and Jordán	2006	Ecological modeling	Physical Effects	No secondary response	Primary	network

Pteropus tonganus	Pacific Flying Fox	Chiroptera	Flying Foxes as Strong Interactors in South Pacific Island Ecosystems: A Conservation Hypothesis	Cox et al.	1991	Conservation Biology	Abundance	Biodiversity	Post-hoc	Determined from literature assessment
Puma concolor	Puma	Carnivora	Summer predation rates on ungulate prey by a large keystone predator: how many ungulates does a large predator kill?	Laundré	2008	Journal of Zoology	Abundance	No secondary response	Primary	Empirical observation or experiment
Pusa hispida	Ringed Seal	Carnivora	Predictions replaced by facts: a keystone species' behavioural responses to declining arctic sea-ice	Hamilton et al.	2015	Biology Letters	Abundance	No secondary response	Post-hoc	Determined from literature assessment
Python molurus bivittatus	Burmese Python	Squamata	Invasive pythons, not anthropogenic stressors, explain the distribution of a keystone species	Sovie et al.	2016	Biological Invasions	Behavioral	No secondary response	Primary	Empirical observation or experiment
Rapana venosa	Veined rapa whelk	Neogastropoda	Bulgarian Initial assessment and GES Report.	BSBD	2013	NA	Abundance	No secondary response	Post-hoc	Determined from literature assessment
Rhyticeros plicatus	Blyth's Hornbill	Bucerotiformes	The Frugivore Community and the Fruiting Plant Flora in a New Guinea Rainforest: Identifying	Mack and Wright	2005	Tropical Fruits and Frugivores	Physical Effects	No secondary response	Primary	network

			Keystone Frugivores							
Sabellaria spinulosa	Ross worm	Sabellariidae	Sabellariaspinulosa reef: a scoring system for evaluating 'reefiness' in the context of the Habitats Directive	Hendrick and Foster-Smith	2006	Journal of the Marine Biological Association of the United Kingdom	Biodiversity	Physical Effects	Post-hoc	Determined from literature assessment
Salangichthys tangkahkeii	Japanese icefish	Osmeriformes	Using a topological approach to identify keystone species of fish in eutrophic lake ecosystems: A case of Zhushan Bay, Taihu Lake	Ren et al.	2022	Fisheries Management and Ecology	Biodiversity	No secondary response	Primary	network
Salminus brasiliensis	Dourado	Characiformes	Climate change will decrease the range of a keystone fish species in La Plata River Basin, South America	Ruaro et al.	2019	Hydrobiologia	Abundance	No secondary response	Post-hoc	Determined from literature assessment
Salmo gairdneri	Rainbow Trout	Salmoniformes	Effects of Fish in River Food Webs	Power	1990	Science	Abundance	No secondary response	Primary	Empirical observation or experiment
Salmo salar	Atlantic Salmon	Salmoniformes	Existing biodiversity, non-indigenious species, food-web and seafloor integrity GEnS indicators. DEVOTES FP7 Project	Teixeira et al.	2014	NA	Abundance	No secondary response	Post-hoc	Determined from literature assessment
Salmo trutta	Brown Trout	Salmoniformes	Existing biodiversity, non-indigenious	Teixeira et al.	2014	NA	Abundance	No secondary response	Post-hoc	Determined from literature assessment

			species, food-web and seafloor integrity GEnS indicators. DEVOTES FP7 Project							
Sander lucioperca	Zander	Perciformes	Existing biodiversity, non-indigenious species, food-web and seafloor integrity GEnS indicators. DEVOTES FP7 Project	Teixeira et al.	2014	NA	Abundance	No secondary response	Post-hoc	Determined from literature assessment
Sarda sarda	Atlantic Bonito	Scombriformes	Overfishing drives a trophic cascade in the Black Sea	Daskalov	2022	Marine Ecology Progress Series	Abundance	No secondary response	Post-hoc	Determined from literature assessment
Scomber scombrus	Atlantic mackerel	Scombriformes	Centennial Decline in the Trophic Level of an Endangered Seabird after Fisheries Decline	Becker and Beissinger	2006	Conservation Biology	Chemical and Energy Cycling	No secondary response	Primary	Empirical observation or experiment
Scophthalmus maximus	Turbot	Pleuronectiformes	The State-of-Art of the Black Sea Turbot Spawning Population off Crimea (1998-2010)	Giragosov and Khanaychenko	2013	Turkish Journal of Fisheries and Aquatic Sciences	Chemical and Energy Cycling	No secondary response	Post-hoc	Determined from literature assessment
Sephanoides sephaniodes	Green-backed firecrown	Apodiformes	Linking Socioeconomics to Biodiversity in the City: The Case of a Migrant Keystone Bird Species	Villaseñor and Escobar	2022	Frontiers in Ecology and Evolution	Biodiversity	No secondary response	Post-hoc	Determined from literature assessment

Sesarma reticulatum	Marsh Crab	Decapoda	Sea-level rise and the emergence of a keystone grazer alter the geomorphic evolution and ecology of southeast US salt marshes	Crotty et al.	2020	Proceedings of the National Academy of Sciences	Abundance	Chemical and Energy Cycling	Primary	Empirical observation or experiment
Sesarma semperi longicristatum	Australasian mangrove crab	Amphionidacea	Keystone species and mangrove forest dynamics: the influence of burrowing by crabs on soil nutrient status and forest productivity	Smith et al.	1991	Estuarine, Coastal and Shelf Science	Chemical and Energy Cycling	No secondary response	Primary	Empirical observation or experiment
Siren intermedia	Lesser Siren	Urodela	Identifying potential keystone species from field data – an example from temporary ponds	Fauth	2002	Ecology Letters	Biodiversity	No secondary response	Primary	Empirical observation or experiment
Solenopsis geminata	Fire ant	Hymenoptera	Effect of a Keystone Predaceous Ant, Solenopsis Geminata, on Arthropods in a Tropical Agroecosystem	Risch and Carroll	1982	Ecology	Abundance	No secondary response	Primary	Empirical observation or experiment
Spermophilus citellus	European Ground Squirrel	Rodentia	Ecological roles and conservation challenges of social, burrowing, herbivorous mammals in the world's grasslands	Davidson et al.	2012	Frontiers in Ecology and the Environment	Physical Effects	No secondary response	Post-hoc	Determined from literature assessment

Sphyrapicus nuchalis	Red-naped Sapsucker	Piciformes	Double keystone bird in a keystone species complex.	Daily et al.	1993	PNAS	Physical Effects	Biodiversity	Post-hoc	Determined from literature assessment
Sphyrapicus varius	Yellow-bellied Sapsucker	Piciformes	Early breeders choose differently – Refining measures of habitat quality for the yellow-bellied sapsucker (Sphyrapicus varius), a keystone species in the mixedwood boreal forest	Squires and Bunnell	2018	PLOS ONE	Biodiversity	Physical Effects	Post-hoc	Determined from literature assessment
Sphyrna lewini	Scalloped Hammerhead	Carcharhiniformes	Cascading Effects of the Loss of Apex Predatory Sharks from a Coastal Ocean	Myers et al.	2007	Science	Abundance	No secondary response	Post-hoc	meta-anlsysis
Sprattus sprattus	European Sprat	Clupeiformes	Human-induced Trophic Cascades and Ecological Regime Shifts in the Baltic Sea	Österblom et al.	2007	Ecosystems	Biodiversity	Chemical and Energy Cycling	Post-hoc	Determined from literature assessment
Squalus acanthias	Spiny Dogfish	Squaliformes	Existing biodiversity, non-indigenious species, food-web and seafloor integrity GEnS indicators. DEVOTES FP7 Project	Teixeira et al.	2014	NA	Abundance	No secondary response	Post-hoc	Determined from literature assessment
Stegastes Fasciolatus	Yelloweye Damselfish	Perciformes	Damselfish as Keystone Species in Reverse: Intermediate Disturbance and	Hixon and Brostoff	1983	Science	Behavioral	Biodiversity	Primary	Empirical observation or experiment

			Diversity of Reef Algae							
Sturnira lilium	Little yellow shouldered Bat	Chiroptera	Keystone species in seed dispersal networks are mainly determined by dietary specialization	Mello et al.	2015	Oikos	Abundance	No secondary response	Primary	network
Sylvilagus palustris	Marsh rabbit	Lagomorpha	Invasive pythons, not anthropogenic stressors, explain the distribution of a keystone species	Sovie et al.	2016	Biological Invasions	Behavioral	No secondary response	Post-hoc	Determined from literature assessment
Theragra chalcogramma	Alaska Pollock	Gadiformes	Anadromous Fish as Keystone Species in Vertebrate Communities	Wilson and Halupka	1995	Conservation Biology	Abundance	No secondary response	Post-hoc	Determined from literature assessment
Thomomys bottae	Botta's Pocket Gopher	Rodentia	Importance of Belowground Herbivory: Pocket Gophers May Limit Aspen to Rock Outcrop Refugia	Cantor and Whitman	1989	Ecology	Abundance	No secondary response	Primary	Empirical observation or experiment
Thunnus albacares	Yellowfin Tuna	Scombriformes	Existing biodiversity, non-indigenious species, food-web and seafloor integrity GEnS indicators. DEVOTES FP7 Project	Teixeira et al.	2014	NA	Abundance	No secondary response	Post-hoc	Determined from literature assessment
Thunnus thynnus	Atlantic Bluefin Tuna	Scombriformes	Existing biodiversity, non-indigenious species, food-web	Teixeira et al.	2014	NA	Abundance	No secondary response	Post-hoc	Determined from literature assessment

			and seafloor integrity GEnS indicators. DEVOTES FP7 Project							
Tursiops truncatus	Common Bottlenose Dolphin	Cetartiodactyla	Overfishing drives a trophic cascade in the Black Sea	Daskalov	2022	Marine Ecology Progress Series	Abundance	No secondary response	Post-hoc	Determined from literature assessment
Tursiops truncatus ponticus	Black Sea Bottlenose Dolphin	Cetartiodactyla	Overfishing drives a trophic cascade in the Black Sea	Daskalov	2022	Marine Ecology Progress Series	Abundance	No secondary response	Post-hoc	Determined from literature assessment
Ultracoelostoma assimile	Sooty Beech Scale	Hemiptera	Beech honeydew: Seasonal variation and use by wasps, honey bees, and other insects	Moller and Tilley	1989	New Zealand Journal of Zoology	Biodiversity	Physical Effects	Primary	Empirical observation or experiment
Ultracoelostoma brittini	Beech Scale	Hemiptera	Beech honeydew: Seasonal variation and use by wasps, honey bees, and other insects	Moller and Tilley	1989	New Zealand Journal of Zoology	Biodiversity	Physical Effects	Primary	Empirical observation or experiment
Uncia uncia	Snow leopard	Carnivora	Restoring a keystone predator may endanger a prey species in a human-altered ecosystem: the return of the snow leopard to Sagarmatha National Park	Lovari et al.	2009	Animal Conservation	Abundance	No secondary response	Primary	Empirical observation or experiment
Upogebia pusilla	Mud shrimp	Eucarida	Bulgarian Initial assessment and GES Report.	BSBD	2013	NA	Abundance	No secondary response	Post-hoc	Determined from literature assessment
Urobatis halleri	Round Stingray	Myliobatiformes	Modeling trophic interactions to	Valls et al.	2012	Marine Ecology	Abundance	No secondary response	Primary	network

			assess the effects of a marine protected area: case study in the NW Mediterranean Sea			Progress Series				
Ursus arctos	Brown Bear	Carnivora	A multidecade experiment shows that fertilization by salmon carcasses enhanced tree growth in the riparian zone	Quinn et al.	2018	Ecology	Chemical and Energy Cycling	Life History	Primary	Empirical observation or experiment
Xenopipo atronitens	Black Manakin	Passeriformes	Black Manakin (Xenopipo atronitens) as a keystone species for seed dispersal in a white-sand vegetation enclave in Southwest Amazonia	Santos et al.	2022	Community Ecology	Abundance	Biodiversity	Primary	network
Xerus inauris	Cape Ground Squirrel	Rodentia	Ecological roles and conservation challenges of social, burrowing, herbivorous mammals in the world's grasslands	Davidson et al.	2014	Frontiers in Ecology and the Environment	Physical Effects	No secondary response	Post-hoc	Determined from literature assessment
